# Acute Compartment Syndrome exists and can lead to irreversible outcomes if not treated in time

**DOI:** 10.1590/0100-6991e-20223350-en

**Published:** 2022-08-25

**Authors:** BRUNO MONTEIRO TAVARES PEREIRA

**Affiliations:** 1 - Universidade Vassouras, PRPPG - Vassouras - RJ - Brasil; 2 - Santa Casa de Campinas, Programa de Residência Médica Cirurgia Geral - Campinas - SP - Brasil; 3 - Instituto Terzius, Diretor Acadêmico - Campinas - SP - Brasil; 4 - Colégio Brasileiro de Cirurgiões, Diretor de Treinamento - Programa A.C.U.T.E. - Rio de Janeiro - RJ - Brasil; 5 - World Society of the Abdominal Compartment, President (2017 - 2019) - Antuerpia - Bélgica

**Keywords:** Intra-Abdominal Hypertension, Acute Disease, General Surgery, Emergency Treatment, Hipertensão Intra-Abdominal, Abdome Agudo, Cirurgia Geral, Tratamento de Emergência

## Abstract

ACS is a potentially lethal condition caused by any event that produces an increase in IAP, inducing systemic collapse, tissue hypoperfusion and organ dysfunction. Thus, ACS is not exclusively a problem of the traumatic and surgical patient population.Iatrogenic ACS predisposes patients to multiple organ failure if no urgent action is taken.

## RESUMO

A SCA é uma condição potencialmente letal causada por qualquer evento que produza aumento da PIA induzindo colapso sistêmico, hipoperfusão tecidual e disfunção orgânica. Assim, a SCA não é um problema exclusivamente da população de pacientes traumáticos e cirúrgicos. A SCA iatrogénica predispõe os pacientes à falência de múltiplos órgãos se nenhuma ação urgente for tomada.

The father of an eight-year-old girl was in my clinic. She died of Abdominal Compartment Syndrome (ACS) resulting from an anesthetic procedure, culminating in gastric perforation and subsequent sudden increase in intra-abdominal pressure (IAP) that was not diagnosed in time to avoid an irreversible outcome.

This case moved me a lot. I have been studying Intra-abdominal Hypertension (IAH) and ACS for 15 years and I wonder why in Brazil we have made so little progress. In the last two years, three iatrogenic cases of acute ACS have come to my attention, resulting from some type of anesthetic-surgical or endoscopic procedure. An average of 1.5/year just in the interior of São Paulo State. This is a small number and perhaps underestimated due to the lack of knowledge on the subject, but with a very high mortality rate. Two of the three patients died. The case of the surviving patient depended exclusively on prior information about ACS by the endoscopist, who was also a surgeon. Noticing the perforation of the colon during colonoscopy, the endoscopist-surgeon performed clipping of the perforation, made direct telephone contact and took the patient to the hospital where I was. The immediate treatment for a patient who exhibited signs of severe shock such as hypotension, tachypnea, diaphoresis, a tense, painful abdomen, and an imminent feeling of death was decompressive abdominal puncture with a 14-gauge Abocath®. As is done in hypertensive pneumothorax, acute Abdominal Compartment Syndrome (hypertensive pneumoperitoneum) requires immediate decompression or may lead to a hopeless scenario. In the patient in question, the response to the puncture was exactly the same in the case of a tension pneumothorax, that is, immediate relief of symptoms and full recovery of the perfusion state. This case was a happy one compared to so many other known and unknown cases.

The question that absolutely needs to be asked to this community of surgeons is: until when? How long are we going to ignore the presence of ACS resulting from diseases, surgical procedures, or iatrogenic events? Awareness and knowledge about ACS urgently need to be present in the day-to-day of interventional physicians.

We know that ACS can present itself in a more insidious (less severe) and more acute (more severe) form and that, without a doubt, the progressive perpetuation of a hypoperfusion state remains associated with the presence of systemic collapse, a good reason for this to serve as a warning to the medical community^1^ ^-^ ^4^.

Evidence is already plenty. The recently published IROI prospective observational study demonstrated that intra-abdominal hypertension occurred in approximately half of all subjects and was twice as prevalent in mechanically ventilated patients as in spontaneously breathing patients. The presence and severity of intra-abdominal hypertension during the observation period significantly and independently increased mortality at 28 and 90 days. Five admission variables were independently associated with the presence or development of intra-abdominal hypertension. Positive fluid balance was associated with the development of intra-abdominal hypertension after the first day. By definition, IAP is the pressure contained in the abdominal compartment. Although from a physiological point of view, IAP can reach transient markers of up to 80mmHg (coughing, Valsalva maneuver, weight lifting, etc.), these values cannot be tolerated for long periods of time, as occurs in iatrogenic ACS. According to the World Abdominal Compartment Society (WSACS), IAH is defined as IAP above 12mmHg on two consecutive measurements within a 4-6 hour interval. However, this concept does not extrapolate to cases of acute ACS, where extremely high and sustained pressure requires immediate intervention. In insidious settings, the harmful effects of IAH occur long before the onset of ACS, and patients who have IAH have an 11-fold increased chance of mortality compared to those who do not have IAH/ACS. In acute ACS, such as iatrogenic, often clinically translated as hypertensive pneumoperitoneum, the rapid progression to ACS defined as an IAP >20mmHg has immediate clinical and hemodynamic consequences. Acute ACS should therefore be seen as the end result of a sudden and sustained rise in IAP leading to imminent risk of death. In these specific cases, the routine measurement of IAP becomes meaningless, as does the request for a chest x-ray for the diagnosis of tension pneumothorax. Common causes of multiple organ dysfunction in the presence of sustained ACS are: metabolic acidosis, oliguria/anuria, elevated airway pressure or hypercarbia refractory to increased respiratory rate, hypoxemia refractory to oxygen therapy and PEEP, and intracranial hypertension. In the case of iatrogenic ACS specifically, systemic collapse is more commonly observed and much more severe due to a sudden and sustained increase in intra-abdominal pressure, decreased venous return, decreased cardiac output, increased intrathoracic pressure, increased ventilatory resistance, increased intracranial pressure, decreased cerebral perfusion pressure, and polycompartmental syndrome^1^ ^-^ ^7^.

The consequences of acute ACS or Acute Abdominal Hypertension Syndrome (AAHS) do not stop there. The duration of ischemia, that is, the duration of the process of exaggerated hypertension in the abdominal compartment, influences the outcome and mortality rates. Patients who remain for an extended period in undiagnosed acute abdominal compartment syndrome are more susceptible to systemic collapse in a short period or subject to ischemia-reperfusion syndrome^8^.

Ischemia-reperfusion injury is a critical condition in which cell damage must be controlled and organ function preserved. Ischemia is simply defined as a consistent tissue hypoperfusion state inducing the anaerobic cell cycle and consequently lower ATP production and lactate production. Various conditions, such as sepsis, acute coronary syndrome, organ transplantation, compartment syndrome itself, and limb injury, are obviously causes of tissue hypoperfusion. Previous studies have demonstrated the importance of reducing hypoperfusion time to preserve organ function. The Surviving Sepsis Campaign guidelines recommend goal-directed early therapy for timely resuscitation, including early antibiotic treatment, and resuscitation with adequate fluids and vasopressors to reduce peripheral tissue hypoperfusion, in other words, treat the cause of the infection and optimize the oxygen transport based on the DO_2_ formula (DO_2_ = DC x CaO_2_). For acute coronary syndrome, in turn, the American Heart Association (AHA) guidelines recommend early revascularization to control myocardial injury and reduce ischemia-reperfusion injury as soon as possible. Similar recommendations have been made for organ transplantation and limb injury. However, no studies exist to date to define how much post-acute ACS reperfusion syndrome can interfere with mortality rates. Clinical signs that it happens are evident, though. Recent studies have shown that reperfusion has the potential to induce subsequent injury to ischemic tissue (ischemia-reperfusion injury), presenting itself as a challenge for the preservation of vital organic functions. Ischemia-reperfusion injury is associated with severe clinical manifestations, including acute heart failure, brain dysfunction, gastrointestinal dysfunction, systemic inflammatory response syndrome, and multiple organ dysfunction syndrome. It is a critical medical condition that represents an important therapeutic challenge that include the involvement of reactive oxygen species (ROS) and cell death pathways. When blood supply is restored after prolonged ischemia, local inflammation and ROS production increase, leading to secondary injury. Cell damage induced by prolonged ischemia-reperfusion injury can lead to apoptosis, autophagy, necrosis, and necroptosis^9^.

Finally, acute ACS or hypertensive pneumoperitoneum is a potentially lethal condition caused by any event that produces an increase in IAP inducing systemic collapse, tissue hypoperfusion, and organ dysfunction. When not treated in time, the devastating clinical conditions of acute ACS lead to systemic collapse; when treated late, they amount to the ischemia-reperfusion syndrome, adding another lethal factor. Acute Abdominal Hypertension Syndrome (AAHS), may be a serious complication of endoscopic, anesthetic or minimally invasive procedures, and predisposes patients to progressive multiple organ failure and high mortality rates if no emergent action is taken. Early diagnosis, based on medical knowledge on the subject, clinical attention, and understanding of risk factors for acute ACS or AAHS is therefore the only viable opportunity for the best and simplest emergency therapy: decompression puncture.
